# Two cases of inflammatory myofibroblastic tumor treated with targeted drugs: A case report

**DOI:** 10.1097/MD.0000000000038136

**Published:** 2024-05-24

**Authors:** Mengyao Liu, Dongyuan Zhu

**Affiliations:** a Rare Tumors Department, Shandong Cancer Hospital and Institute, Shandong First Medical University and Shandong Academy of Medical Sciences, Jinan, China.

**Keywords:** ALK, IMT, ROS1, targeted therapy

## Abstract

**Introduction:**

Inflammatory myofibroblastic tumor (IMT) is a rare invasive soft tissue tumor. Many IMTs are positive for anaplastic lymphoma kinase (ALK) with ALK gene fusion; other gene mutations have also been reported, which indicates a key role for genetic testing and the development of target therapy to optimize treatment strategies.

**Patient concerns:**

We report 2 patients who obtained clinical benefits following targeted treatment with ensartinib.

**Diagnosis:**

The first patient was diagnosed as IMT, with TFG-ROS1 fusion gene mutation. The second patient was IMT harboring the ALK-STRN fusion gene mutation.

**Interventions:**

We performed gene testing for these 2 patients. According to the test result, both patients received ensartinib 225 mg QD as targeted therapy for a 30-day cycle.

**Outcomes:**

The first patient achieved partial remission and maintained a stable state for 14.7 months. The second patient was treated for 10 months and reached complete remission after 5 months and is currently still benefiting from treatment. Treatment-related side effects were mild in both patients.

**Conclusion:**

Our cases provided some new insights and approaches for the clinical diagnosis and treatment of IMT.

## 1. Introduction

Inflammatory myofibroblastic tumor (IMT) is a mesenchymal soft tissue tumor that can arise at multiple anatomic sites and can occur at any age, especially showing a predilection for the lung, pelvis, or retroperitoneum of children and young adults.^[1,2]^ The histological characteristics of IMTs are distinctively composed of myofibroblastic-type cells and include spindle cell proliferation with inflammatory infiltrate.^[3]^ Next-generation sequencing (NGS) of RNA shows that 85% of cases harbored kinase gene fusions, involving ALK, ROS1, or PDGFRβ, in addition, RET rearrangement and overexpression of NTRK3 have also been reported.^[4–6]^ Approximately 50% of IMT express ALK mutations, the most common involve chromosomal rearrangements, although the expression of fusion genes has also been reported retrospectively.^[7]^

Currently, surgical resection remains the main treatment method for IMT. However, treatment options for patients with unresectable and/or advanced diseases are limited. Targeted therapy is an important treatment option for this population and patients may benefit from drugs targeting the aforementioned gene mutations. The US Food and Drug Administration has recommended crizotinib for the treatment of ALK fusion-positive lung cancer and IMT. ROS1 is evolutionarily related to ALK and shares 49% amino acid sequence homology with ALK in the kinase domains. In recent years, ROS1 has received increasing attention and has been detected in a variety of tumors.^[8]^ Several ALK inhibitors have demonstrated in vitro inhibitory activity against ROS1.^[9]^ ALK inhibitors have recently entered clinical trials. Ensartinib is a new oral, second-generation small-molecule drug, which is a strong and highly selective ALK-tyrosine kinase inhibitor (TKI).^[10,11]^ Herein, we present 2 cases of patients with IMT receiving ensartinib as targeted treatment who obtained clinical benefits. Our cases provide novel ideas and approaches for clinical diagnosis and treatment.

## 2. Case reports

### 2.1. Case 1

A 37-year-old young woman, whose medical history and family history were not remarkable, presented with vomiting without obvious cause from November 2016. An intragastric mass was identified by gastroscopy examination. In December 2016, she received a laparoscopic subtotal gastrectomy under general anesthesia. The postoperative pathological diagnosis was undifferentiated spindle cell sarcoma and IMT was considered. The postoperative follow-up was conducted regularly. A computed tomography (CT) scan was performed in July 2019 and an intra-abdominal mast was detected, which was considered a recurrence. On September 9, 2019, under general anesthesia, she underwent surgical intervention that involved resection of a retroperitoneal mass, total gastrectomy, pancreatic body and tail, spleen, partial left colon, and lymph node. The postoperative pathological diagnosis was consistent with IMT. Both fluorescence in situ Hybridization and Immunohistochemistry (IHC) were ALK-negative. Gene testing using the second surgical specimen showed that ALK rearrangement was not detected and that fusion mutations were absent. Programmed cell death ligand-1 tumor proportion score was 5%. Systemic treatment was not performed after the second surgery.

On August 11, 2020, a reexamination of the positron emission tomography (PET)-CT showed that after the second gastritis myofibroblastoma surgery, there were soft tissue nodules on both sides of the uterine body, and there was a high possibility of metastasis. Up to this time, the patient’s general condition was still good, but she reported occasional stomach discomfort, and since gastric surgery in 2019, her blood tests all showed anemia, with a hemoglobin minimum around 70 g/L. Other laboratory tests were normal.

**Figure 1.**
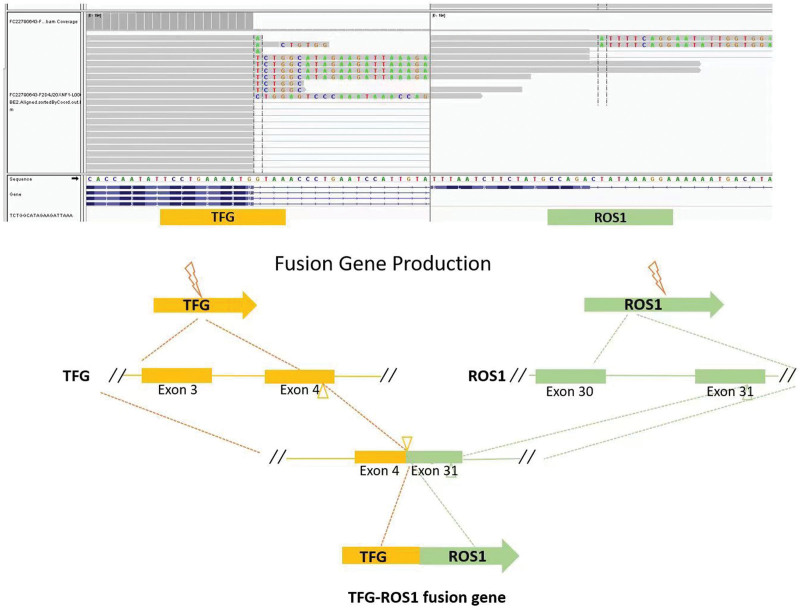
The gene fragment analysis and schematic diagram of TGF-ROS1 fusion (case 1).

As the patient had experienced 2 operations and there were multiple recurrences and metastases, the patient was in an advanced stage, and was no longer eligible for surgical treatment. The patient’s next treatment mainly involved medical treatment. The patient received treatment with arotinib, a small-molecule domestic multitarget antiangiogenic agent, on August 28, 2020. The drug was administered 12 mg daily for 2 weeks and then stopped for 1 week. This patient developed severe aphthous stomatitis 6 months after the start of treatment. Through symptomatic treatment, including the use of mouthwash and oral gel medicine, the patient’s symptoms were gradually alleviated. After nearly 2 years of treatment, the subcutaneous mass in the lower left abdomen gradually increased. We performed a puncture and obtained pathology specimens in June 2022. Subsequently, NGS was repeated. Unlike the first study, RNA fusion gene testing and genomic profiling identified the presence of a ROS1 kinase fusion in this patient, the mutant type was a TFG exon4 and ROS1 exon35 gene fusion, with a mutation frequency of 33.3% (Fig. 1).

**Figure 2.**
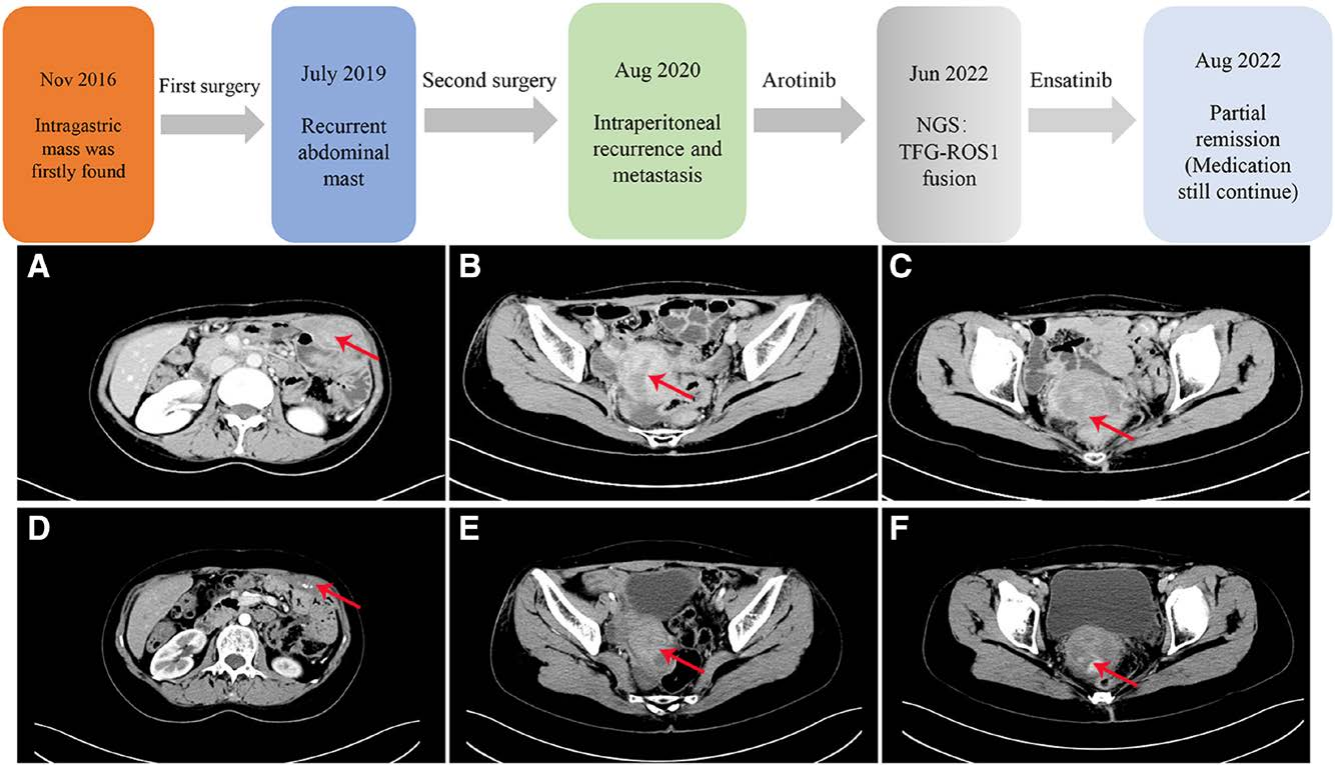
Treatment timeline and comparison of images before and after ensartinib treated 2 mo of 3 evaluated lesions (case 1). (A) Left anterior upper abdominal lesion before treatment; (B) lesion in the right rear of cervix before treatment; (C) lesion behind the cervix before treatment; (D) left anterior upper abdominal lesion after treatment; (E) lesion in the right rear of cervix after treatment; (F) lesion behind the cervix after treatment.

Due to the limited economic conditions of this patient, her family could not afford to pay the high cost of treatment. Considering the patient’s financial concerns and based on the molecular findings, she was treated with the ROS1 TKI, ensartinib 225 mg QD, for a 30-day cycle. She provided signed informed consent prior to treatment. She was followed up by CT every 2 months. She presented remission of paraneoplastic anemia and symptomatic improvement and the hemoglobin level increased 80 to 90 g/L. After 2 months of treatment, the lesion was partially reduced and the efficacy was evaluated as partial remission according to the Response Evaluation Criteria in Solid Tumors v.1.1 (Fig. 2). On subsequent treatment, the patient’s condition remained stable. The main side effects included a slight increase in transaminase levels, which was cured after administering liver protective drugs.

### 2.2. Case 2

A 59-year-old middle-aged man, with normal medical history and family history, was diagnosed with a “gastric tumor” and underwent a partial laparoscopic gastrectomy in September 2021. The postoperative pathological diagnosis showed an epithelioid inflammatory myofibroblastic sarcoma, involving gastric mucosa, vascular tumor thrombus, nerve invasion, and Ki67+ of 26%. The patient received chemotherapy with paclitaxel plus cisplatin for 6 cycles and maintenance treatment with nedaplatin for 4 cycles.

**Figure 3.**
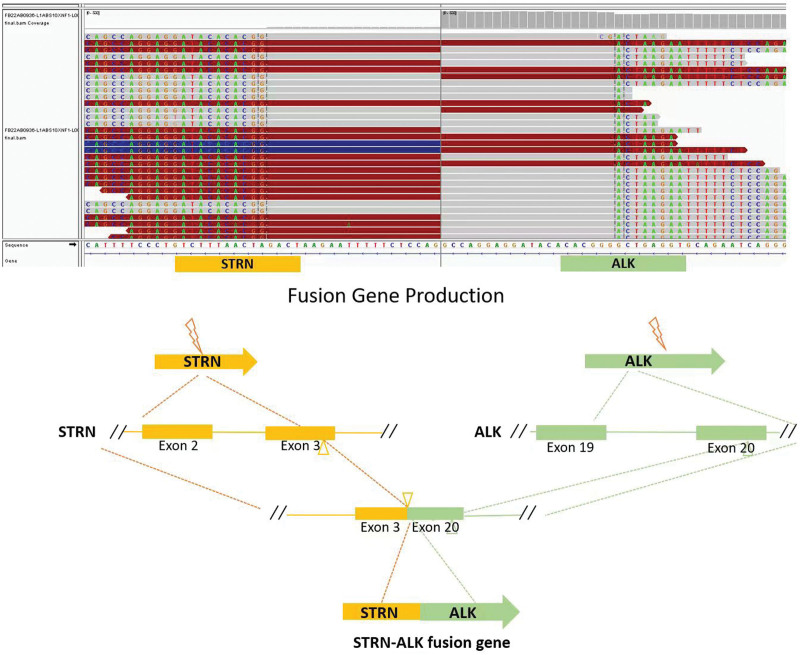
The gene fragment analysis and schematic diagram of STRN-ALK fusion (case 2). ALK = anaplastic lymphoma kinase.

The patient came to our hospital for the first time in November 2022. A reexamination of PET/CT was performed on November 2, 2022, there was no abnormal high metabolism at the anastomotic site. Postoperatively, multiple hypermetabolic peritoneal metastases were detected with perisplenic lesions involving the diaphragm. The pathology diagnosis based on the abdominal mass puncture biopsy was obtained on November 7, 2022, the results were as follows: malignant tumor, consistent with inflammatory myofibroblastic sarcoma. IHC showed ALKp80(+). To further clarify the diagnosis and to explore treatment options, genetic tests were conducted using postoperative bioptic samples, and NGS revealed ALK exon20 and STRN exon3 fusion mutation, with mutation abundance of 32.31% (Fig. 3).

Based on the pathology findings, imaging examination, and genetic testing, a diagnosis of IMT was relatively clear and a late-stage tumor was defined. The patient had no other symptoms of discomfort except occasional right upper abdominal discomfort. The patient’s general condition was good and all test indexes were normal.

**Figure 4.**
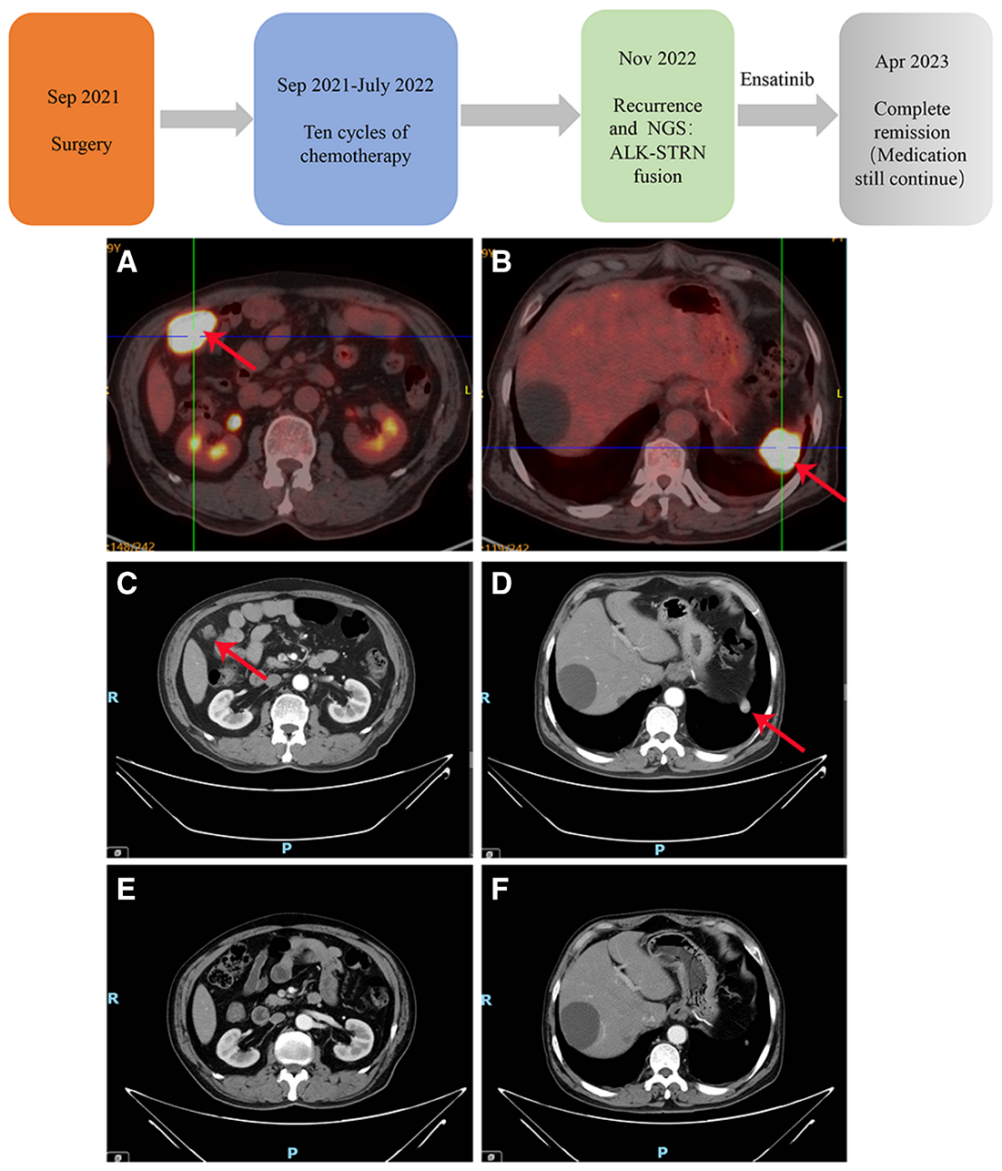
Treatment timeline and comparison of images before and after ensartinib treated 5 mo of 2 evaluated lesions (case 2). (A) Lesion in the right lower abdomen before treatment; (B) lesion in the left lower abdomen before treatment; (C) Lesion in the right lower abdomen after treatment of 2 mo; (D) lesion in the left lower abdomen after treatment of 2 mo; (E) lesion in the right lower abdomen after treatment of 5 mo; (F) lesion in the left lower abdomen after treatment of 5 mo.

Subsequently, the patient received ensartinib 225 mg QD as targeted therapy for a 30-day cycle. He provided signed informed consent prior to treatment too. The patient was reviewed with an enhanced CT scan approximately every 2 months to assess response to treatment. After 2 months of treatment, partial remission was achieved, and at 5 months of treatment, complete remission was observed which is still maintained now (Fig. 4).

There were no major discomfort complaints and no other drug-related side effects in the above 2 cases, and routine blood examination and liver and kidney function were normal every time, suggesting the good clinical efficacy of ensartinib. Ensartinib-targeted therapy is continued and regular outpatient follow-up is continued.

## 3. Discussion

Positive expression of ALK and ROS1 are detected in 61.1% and 5.6%, respectively, of patients with IMT. ALK-negative IMTs may present ROS1 rearrangement or other mutations in gene loci as a possible oncogenic mechanism, the detection of these alterations may be of diagnostic value and be helpful in determining promising therapeutic strategies.^[4]^

Ensartinib is a second-generation ALK inhibitor independently developed in China. In the eXalt3 randomized clinical trial, ensartinib was evaluated as a new first-line treatment option for patients with ALK-positive nonsmall cell lung cancer (NSCLC). The median progression-free survival (mPFS) of the ensartinib group was significantly longer than that of the crizotinib group (25.8 vs 12.7 months; log-rank *P* < .001), meanwhile, treatment-related toxicity was well tolerated, achieving excellent efficacy in the ensartinib treatment group without the expense of safety.^[12]^ Furthermore, in tumors with ALK mutations other than lung cancer, crizotinib is not covered by medical insurance in the Chinese mainland, although ensartinib is also self-funded, the monthly cost of crizotinib treatment is nearly 3 times that of ensartinib. For most patients, the latter is more acceptable and has little economic pressure.

The activity of cancer-specific ALK variants is required for tumor maintenance. Multiple preclinical studies have shown that specific small-molecule ALK TKIs can delay tumor growth and/or induce tumor regression in xenograft and transgenic models.^[10]^ However, few reports are available on the efficacy of ensartinib treatment for metastasis IMT, with no consistent prediction of the efficacy of TKI in metastatic IMT. Herein, we describe 2 cases with IMT that received ensartinib and achieved clinical benefits after treatment.

In the first case, the patient had been on treatment for nearly 7 years since her diagnosis in 2016. In the early tumor stage, surgery is the main treatment option, and in the later stages, inoperable patients may benefit from targeted therapy. In this patient, the associated mutations were initially not detected on genetic testing. Considering that IMT is not sensitive to chemotherapy and may benefit from targeted therapy, we chose the multitargeted drug allotinib as treatment. The patient remained stable for about 22 months. Subsequently, the disease progressed and a puncture was obtained, genetic testing was then performed, from which the ROS1 mutation was found. This finding provided a new option for subsequent treatment. The patient was treated with ROS1 inhibitor ensartinib. The lesion achieved partial remission. And the tumor mass maintains a stable state for 14.7 months. To our knowledge, this is the first case of ROS1 fusion showing that the use of ensartinib as TKI in IMT has clinical benefits.

In the second case, the patient had the ALK-STRN fusion mutation and was treated with ensartinib. Tumor lesions continued to shrink and eventually reached complete remission, which is still maintained today. It has been 10 months since the initial treatment and the patient is in good condition now.

In IMT, ALK-positive cases show a good prognosis. In contrast, negative ALK may be more aggressive and may be associated with a higher incidence of metastases. These features may be attributed to the fact that the ALK-negative neoplasm indicates different genetic abnormalities or entities.^[3,13]^

Patients with ALK-positive and ALK-negative advanced/metastatic IMT were included in the EORTC 90101 clinical trial and received 250 mg of oral crizotinib twice a day. The mPFS of patients with ALK-positive and ALK-negative IMT was 18.0 months and 14.3 months, respectively.^[12]^ Data from our study did not show any significant differences in outcome in ALK-negative patients using ensartinib compared with crizotinib, and for ALK-positive patients, a definitive conclusion has not yet been reached but is worth waiting for.

In both of our patients, the side effects of ensartinib were tolerable. As the efficacy of the treatment was good, the side effects were modest and economically acceptable. The patients have been adhering to the treatment without interruption; the compliance is very good. Ensartinib is beneficial to improve patient prognosis and its specific therapeutic efficacy can be more clearly established in studies with larger sample sizes and in clinical trials.

It is worth mentioning that the critical component for successful selection of this therapeutic approach is the incorporation of highly sensitive NGS testing using platforms capable of detecting both known and novel fusions in multiple oncogenes from a single tumor specimen. Gene mutation screening covered 104 gene exons, fusion-related intron regions, and variable shear regions. The reference genome was GRCh37/hg19. The detection results included point mutations, small fragment insertion and deletion mutations, gene fusion, and variation in the copy number in the coverage area. NGS by RNA has certain advantages in detecting gene mutation and fusion genes. There are no introns at the RNA level, so the detection costs are reduced and mutations are easier to detect. Some complex structural variations at the DNA level, such as gene spacer fusion and 3-gene fusion, are not expressed at the transcriptome level, so detection at the RNA level is more accurate.

The 2 cases prompted us to perform genomic analysis on a larger series of this rare tumor, and targeted treatment not only provided information on this rare tumor type but also offered a rationale for targeted therapeutic strategies with existing TKIs based on the genomic profile of the tumor.

## 4. Conclusions

Herein, we presented 2 cases of advanced/metastatic IMT in ALK-positive and negative patients who were treated with ensartinib and achieved good therapeutic effects in both cases. From these 2 patients, it can be seen that patients with IMT have a good prognosis and achieved longer survival after receiving the appropriate treatment. Genetic testing was performed, including RNA sequencing, and suitable targeted drugs were identified. Targeted therapy is an important treatment option for inoperable patients with IMT. Our cases may provide some helpful insight into the role of precision diagnostics, targeted therapy, and postintervention assessment of patients with IMT harboring ALK and ROS1 or other gene fusion.

A limitation of our study is that there are individual differences in case reports, so the cases were not exactly representative. Therefore, clinical trials with a larger number of participants are needed in the future to clarify the role of targeted therapy for patients with IMT.

## Acknowledgments

All authors thank their companions for their understanding and support in this work.

## Author contributions

**Writing – original draft:** Mengyao Liu.

**Writing – review & editing:** Dongyuan Zhu.
